# Circadian Misalignment and Metabolic Disorders: A Story of Twisted Clocks

**DOI:** 10.3390/biology10030207

**Published:** 2021-03-10

**Authors:** Aurore Woller, Didier Gonze

**Affiliations:** 1 Department of Molecular Cell Biology, Weizmann Institute of Science, Rehovot 76100, Israel; apolaire@gmail.com; 2 Unité de Chronobiologie Théorique, Faculté des Sciences CP 231, Université Libre de Bruxelles, Bvd du Triomphe, 1050 Bruxelles, Belgium

**Keywords:** circadian oscillations, gene regulatory network, light, food, conflicting zeitgebers, computational modeling

## Abstract

**Simple Summary:**

In mammals, many physiological processes follow a 24 h rhythmic pattern. These rhythms are governed by a complex network of circadian clocks, which perceives external time cues (notably light and nutrients) and adjusts the timing of metabolic and physiological functions to allow a proper adaptation of the organism to the daily changes in the environmental conditions. Circadian rhythms originate at the cellular level through a transcriptional–translational regulatory network involving a handful of clock genes. In this review, we show how adverse effects caused by ill-timed feeding or jet lag can lead to a dysregulation of this genetic clockwork, which in turn results in altered metabolic regulation and possibly in diseases. We also show how computational modeling can complement experimental observations to understand the design of the clockwork and the onset of metabolic disorders.

**Abstract:**

Biological clocks are cell-autonomous oscillators that can be entrained by periodic environmental cues. This allows organisms to anticipate predictable daily environmental changes and, thereby, to partition physiological processes into appropriate phases with respect to these changing external conditions. Nowadays our 24/7 society challenges this delicate equilibrium. Indeed, many studies suggest that perturbations such as chronic jet lag, ill-timed eating patterns, or shift work increase the susceptibility to cardiometabolic disorders, diabetes, and cancers. However the underlying mechanisms are still poorly understood. A deeper understanding of this complex, dynamic system requires a global holistic approach for which mathematical modeling can be highly beneficial. In this review, we summarize several experimental works pertaining to the effect of adverse conditions on clock gene expression and on physiology, and we show how computational models can bring interesting insights into the links between circadian misalignment and metabolic diseases.

## 1. Introduction

In mammals a large range of physiological and metabolic processes display a 24 h rhythmic pattern. These processes are controlled by an elaborate circadian system consisting of a finely tuned network of clocks throughout the body coupled together by periodically released hormones, nutrient sensors, and neuronal connections. Besides controlling the sleep–wake cycle, this system appears to be optimized to ensure metabolic homeostasis (by allowing anticipation of food uptake), an effective immune response, and synchronized cell division [1,2]. It relies on the alignment of the light–dark cycle and the feeding–fasting pattern and on a precise genetic clockwork. Dysregulation of this system, through genetic mutations or through alteration of the timing of zeitgebers (jet lag, shift work, ill-timed food intake, etc.) can lead to metabolic syndromes and pathologies, including obesity, diabetes, auto-immunity, and tumorigenesis and cancer [2,3]. By “zeitgeber” we refer to any periodic input that entrains the circadian clock, with light and food being external zeitgebers, while hormones and neuroal projections are internal zeitgebers. 

Understanding the cogs and wheels of this multi-oscillatory system is challenging because it involves the integration of organ-specific genetic control, metabolic regulation, and hormone-mediated inter-organ coupling. Experimentally, the logic of this regulatory system can be probed by genetic studies and by artificial environmental perturbations (e.g., conflicting zeitgebers) and by looking at the consequences on gene expression and on physiology. Experimental studies can be complemented by mathematical modeling investigations. Theoretical models aim at providing a rational explanation for observed dynamic responses and allow the integration of disparate experimental observations. 

We review here several studies that contributed to unraveling the complex interplay between the genetic clocks and the physiology and show how mathematical models help to interpret the observations and to provide guidelines for future investigations. Particular attention is given to the importance of the timing of clock gene expression for proper coordination of metabolism and to the alteration of this timing due to conflicting environmental signals. We first describe the global circadian organization and the genetic circuitry underlying circadian oscillations, which is responsible for the proper timing of gene expression in physiological conditions. We then show, through a selection of experimental and theoretical papers, how the phase relationships between the clock gene expression are altered under ill-timed feeding or in response to a jet lag and how, in turn, those “twisted clocks” impact clock-controlled cellular processes. 

## 2. Global Organization of the Circadian System

The circadian system consists of a fine-tuned network of clocks throughout the body [4]. The suprachiasmatic nucleus (SCN) of the hypothalamus is regarded as the circadian pacemaker [5]. The SCN is composed of two clusters of about 20,000 densely connected neurons in rodents (and about 100,000 neurons in humans). Each neuron expresses a dozen clock genes, which form a transcriptional regulatory network able to generate circadian oscillations, which persist in constant environmental conditions. Although oscillations observed in isolated cells are noisy and present large cell–cell variability, coupling through the circadian release of neuropeptides allows a robust synchronization of the cells [6,7]. The SCN receives light signals from the retina through the retinohypothalamic tract (RHT). Information on the light/dark alternation is thereby transmitted to the hypothalamus and, in normal conditions, entrains the SCN circadian pacemaker. The SCN dictates daily changes in body temperature, determines food intake, controls sleep–wake cycles [8] and, through the control of hormonal release, influences the rhythms in peripheral organs [9,10,11]. For example, by stimulating the secretion of cortisol—the main glucocorticoid (GC) in humans—by the adrenal glands via the hypothalamic–pituitary–adrenal axis, the SCN affects insulin signaling and insulin secretion in the pancreas [12] as well as gluconeogenesis in the liver [13]. This level of control adds to the nutrient-induced secretion of insulin and to the SCN input via the vagus nerve. The SCN also controls the nocturnal release of melatonin by the pineal gland. This hormone may target the SCN but appears to also be involved in the regulation of glucose metabolism [14]. 

Peripheral clocks are present in many organs including the liver, pancreas, gut, adipocytes, or muscles. They all possess autonomous clocks, which were initially viewed as slave oscillators predominantly controlled by the SCN pacemaker [15]. This hierarchical organization of circadian clocks was supported by the observation that the rhythmicity in peripheral organs is lost upon SCN lesion and by SCN transplantation experiments, which showed that host hamster acquired the period of activity from the SCN donors [16,17]. The SCN was then seen as a synchronizing center to keep peripheral clocks in phase [18]. This master–slave view has been challenged by several observations. First, the production of hormones such as melatonin or cortisol does not adjust to the rhythm of the grafted SCN in hamsters [19]. Second, the liver was shown to strongly respond to food intake. An inversion of the food cycle was indeed shown to invert the rhythm of the liver [20,21], leading to a (partial) decoupling of this peripheral clock from the SCN. Third, these peripheral clocks feed back to the SCN. For example, leptin, an appetite suppressor, which is secreted by adipocytes, may target the SCN via the arcuate nucleus (ARC) as demonstrated by the disruption of the circadian rhythm in food intake induced by ablation of leptin-receptor-expressing neurons in the ARC [22]. 

**Figure 1:**
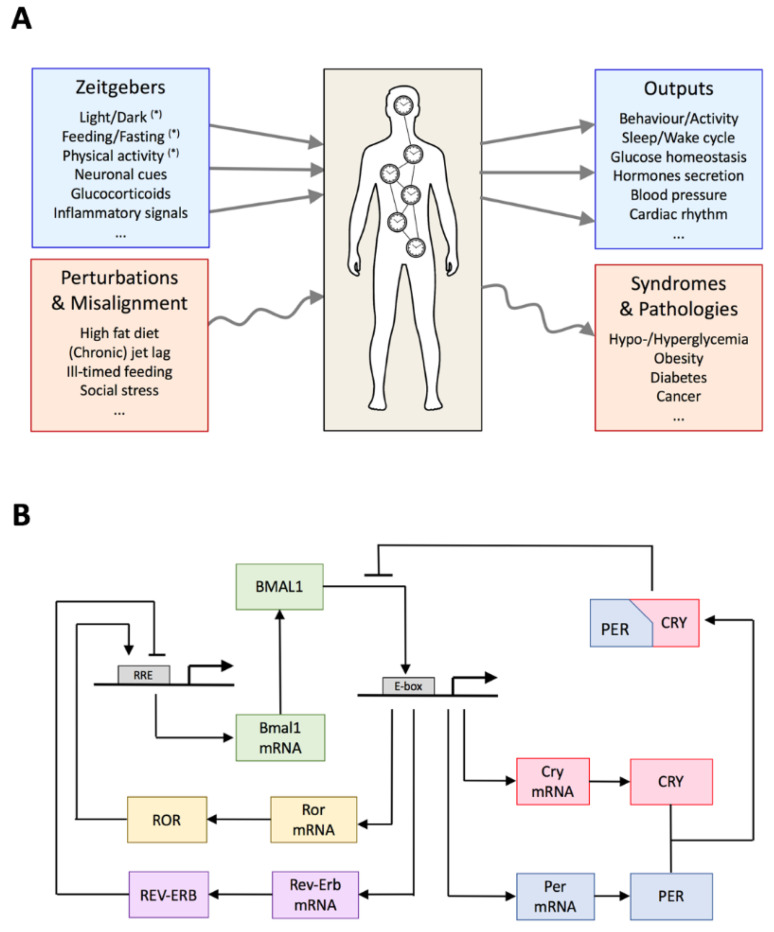
(**A**) The circadian system consists of a network of coupled clocks, receives multiple inputs (zeitgebers), and controls many aspects of physiology and metabolism. Alteration of the zeitgebers (e.g., via circadian misalignment or jet lag) can lead to metabolic syndromes and pathologies. Of note, central and peripheral clocks differ by the nature and/or sensitivity of the different zeitgebers. We can also distinguish external zeitgebers (such as light and food, denoted by an asterisk) and internal zeitgebers (hormones and neural projections involved in the internal coupling between the clocks). (**B**) Scheme of the genetic regulatory network underlying circadian gene expression. The scheme focuses on the core genes/proteins which are circadianly expressed. Clock, which is constitutively expressed, is not represented.

Whereas the SCN mainly senses light, peripheral clocks receive inputs from nutrient sensors, including SIRT1 (Sirtuin 1) [23,24], AMPK [25], hormones [18,26], and neuronal cues [27]. Peripheral clocks are thus subject to multiple zeitgebers, mutually coupled, and able to feed back to the SCN pacemaker. Moreover, central and peripheral clocks differ in their sensitivity for different zeitgebers, and most likely the peripheral clocks themselves also differ in their sensitivity for different zeitgebers. For example, the nutrient sensor AMPK differentially affects different peripheral clocks [28]. As a consequence, changing the timing of one zeitgeber can be sufficient to disorganize the circadian system, since such a shift will have different consequences for the different clocks. Altering zeitgebers thus has effects on the global circadian organization, both at the genetic and at the organ levels, with potential consequences on physiology and behavior (Figure 1A). Understanding the working of this system and the emergence of pathologies thus requires system-level investigations that integrate both genetic and tissue levels.

## 3. Architecture of the Circadian Gene Regulatory Network

The molecular mechanism of the circadian clock in mammals relies on multiple interlocked transcription/translation feedback loops, where several clock genes and regulatory proteins play key roles (see [29] or [30] for comprehensive reviews) (Figure 1B). At the core of this genetic regulatory network, a negative feedback loop is centered on the PER and CRY proteins, which repress the expression of their own genes, *Per* (*period*) and *Cry* (*cryptochromes*). There are actually three homologs of *Per* genes (named *Per1*, *Per2*, and *Per3*) and two *Cry* homologs (*Cry1* and *Cry2*). PER and CRY proteins heterodimerize in the cytoplasm and translocate into the nucleus where they interact and thereby inhibit their transcriptional factor CLOCK:BMAL1. The latter is a heterodimeric complex between CLOCK, encoded by the constitutively expressed *Clock* (*circadian locomotor output cycles kaput*) gene, and BMAL1, encoded by the circadian expression of *Bmal1* (*brain and muscle ARNT-like 1)* gene. PER and CRY proteins are degraded through ubiquitin-dependent pathways, leading to a release of CLOCK:BMAL1, which can then launch a new circadian cycle.

Kinases and phosphatases regulate the phosphorylation state and thereby the localization and stability of the clock proteins [29,30,31]. These multiple phosphorylations contribute to create a delay necessary to produce long-period oscillations and to the fine-tuning of the circadian oscillator [32]. Impaired phosphorylation, due to mutations either in the phosphorylation site of the PER proteins or in their kinases/phosphatases has been associated with advanced/delayed sleep phase syndrome in humans [33] as well as with perturbed feeding rhythms and the development of obesity in mice [34].

Two secondary feedback loops are mediated through transcriptional activation of *Bmal1* by the retinoid-related orphan receptors (RORα,β,γ) [35] and repression by Rev-Erbα/β [36] (Figure 1B). These interlocked regulatory loops contribute to produce a robust rhythm in *Bmal1* transcription. Whereas knockout of *Bmal1* leads to arrhythmicity [37], rhythmic change in BMAL1 abundance is however not required to drive circadian oscillations [38]. The idea that interlocked feedback loops promote and amplify genetic oscillations was also supported by mathematical modeling [39,40]. Moreover, the ROR/Rev-Erb feedback loops induce a delay in *Cry1* expression, which is crucial for proper circadian timing [29,41]. Next to ROR and Rev-Erb, the transcription factors DBP (D-box binding protein) and E4BP4 (E4-binding Protein 4) further participate in circadian gene regulation through binding to D boxes present in the promoter of *Per* and *RevErb* genes [42]. Interestingly, these factors also play an important role in metabolism and immune response regulation [43,44].

This intricate gene regulatory network operates at the single-cell level both in the SCN and in peripheral tissues [45]. In the SCN, there is, however, an additional level of organization. Neurons are indeed strongly coupled together via the circadian production of neurotransmitters and of their receptors [46]. Supported by theoretical models [47,48], it was suggested that this intercellular coupling contributes to generate a robust, overt rhythm from otherwise weak and possibly damped single-cell SCN oscillators [49]. Intercellular coupling appears weak or absent in peripheral organs. Cellular oscillators in these organs, thus, appear mainly under the influence of external cues, which may play the role of global synchronizers. 

Light signals perceived by the retina interact with the SCN clock by inducing the expression of *Per1* and *Per2*, as well as that of *Cry1* and *Cry2* genes [50,51,52]. The effect of light is mediated by CREB, a Ca^2+^/cAMP response element-binding protein [53,54,55]. Phosphorylation of CREB can be induced by light or glutamate and is required for the entrainment of the circadian oscillations by light–dark cycles [53]. Peripheral clocks do not perceive light but are targets of hormonal and neuronal signals coming from the SCN or other peripheral organs. These signals modify clock gene expression. For example, glucocorticoid signaling is known to act on the transcription of *Per* [18] and of *RevErb*α genes [56].

The CLOCK:BMAL1 complex not only activates the transcription of the *Per* and *Cry* genes but also drives the expression of numerous clock-controlled output genes [57]. By recruiting histone-modifying enzymes (through methylation and acetylation), this complex is responsible for chromatin modification, allowing or preventing gene expression [58,59]. BMAL1 targets were highly enriched in genes involved in carbohydrate and lipid metabolism, as well as in genes coding for transcription factors, in particular nuclear receptors [60]. Thus, by stimulating the expression of the PAR-bZIP transcription factors, CLOCK:BMAL1 also controls the expression of many additional genes [61]. Besides its key role in the circadian clock, the transcriptional repressor RevErbα also controls the expression of many genes involved in carbohydrate and lipid metabolism, in the inflammation response, and in hormone signaling [62]. 

## 4. Circadian Timing of Clock Genes and Clock-Controlled Genes in Physiological Conditions

The above gene regulatory network contains all the necessary elements to produce autonomous 24 h oscillations. In particular, the core negative transcriptional feedback, the delay (due to phosphorylations and transport), and several sources of nonlinearity (resulting from transcriptional repression, multisite phosphorylation, and/or complex formation) are key ingredients for the oscillatory mechanism. Moreover, computational simulation shows that these oscillations can readily be entrained by light–dark cycles through the periodic forcing of light-controlled kinetic parameters [39,63,64,65]. 

The occurrence of oscillations is however not the only requirement for a functional clock. The timing of the oscillations (i.e., the phase of each component) is of course critical to guarantee a proper adaptation to the periodic lighting and feeding conditions. Despite the conservation of the genetic machinery in most tissues and a relatively well-conserved entrainment phase of the different clock components [57,66,67], at least under healthy conditions, the phase of numerous clock-controlled genes appears tissue-specific [57,68]. It should also be stressed that the number of oscillatory genes strongly depends on the organ, with the liver ranked at the top with more than 3000 oscillatory genes, and that the number of genes displaying oscillations in several organs is very low [57]. Thus, the circadian scheduling of physiology is highly organ specific. 

It is possible that the multiplicity of feedback loops and apparent redundancy of core clock genes—not absolutely required to generate entrainable circadian oscillations—arose from the need to precisely and robustly set the phase of each component. Gene-specific regulation of post-translational processes may further contribute to adjust the phase of expression of core genes [32]. This complexity also offers more flexibility to the clock, which can be optimized for specific functions in each organ. The difference in the timing of the expression of clock genes and clock-controlled genes across organs can be explained, at least partly, by differences in kinetic parameters and by the combinatorial presence and interplay of multiple regulatory binding sites [69,70]. It is also likely that additional factors such as tissue-specific clock inputs and differentially expressed regulatory genes also contribute to this variability. 

The computational studies mentioned above, together with other theoretical works, support the idea that the clock has evolved to precisely adjust gene expression to cyclical changes in the zeitgebers under natural conditions. As we will see below, the phase of the clock and clock-controlled genes can, however, be perturbed under unusual living conditions and in particular under conflicting time cues such as ill-timed feeding, with consequences on the physiology. 

## 5. Ill-Timed Feeding Pattern and Twisted Clocks

The circadian system allows the organisms to adapt to normal environmental and feeding conditions by anticipating daily changes and, thus, helping them to remain in a homeostatic, healthy state. The proper working of the circadian system, however, relies on the alignment between the environmental lighting conditions and feeding time. By “alignment” we mean that the timings of these external cues are such that the circadian clock system can anticipate these changes and, thereby, guarantee an optimal adaptation. For example, by regulating the transcription of genes involved in insulin secretion, the pancreas prevents an excessive accumulation of blood glucose [71]. The liver also plays a critical role in the maintenance of glucose homeostasis through the circadian regulation of gluconeogenesis [13,72]. There is an increasing amount of evidence showing that circadian disturbances lead to metabolic syndromes and pathologies such as diabetes in humans. These disturbances include shift work, wrong eating timing, sleep loss, and chronic jet lag [73,74,75,76,77,78]. 

To understand how metabolic syndromes and diseases occur under perturbed conditions, it is necessary to investigate the impact of environmental factors and their timing on the clock gene expression and, subsequently, on the physiology. It was, for example, observed that perturbing the metabolism through a high-fat diet or through ill-timed feeding results in tissue and gene-specific alteration of the expression levels of clock and clock-controlled genes, hormone production, and activity [79,80,81,82].

Bray et al. [80] measured clock gene expression in various organs in mice with day- and night-restricted feeding (liver, epididymal fat, gastrocnemius muscle, and heart). They report dramatic phase shifts, in the range of 6 to 11 h, in the expression of clock genes in the liver of mice fed during the day vs. those fed at night. Compared to the liver, restricted feeding had markedly lower and less consistent effects on the phases of clock gene oscillations in epididymal fat, muscle, and heart. In these organs, average phase shifts were around 3 to 7 h. Furthermore, the amplitude of gene expression oscillations were often significantly dampened in these peripheral tissues. Bray et al. [80] also recorded the expression of numerous metabolic genes. Consistently with the behavior of clock genes’ expression, metabolic genes’ expression appears to be strongly rhythmic and shifted by 12 h in the liver and more damped and only partially shifted in other organs.

**Figure 2:**
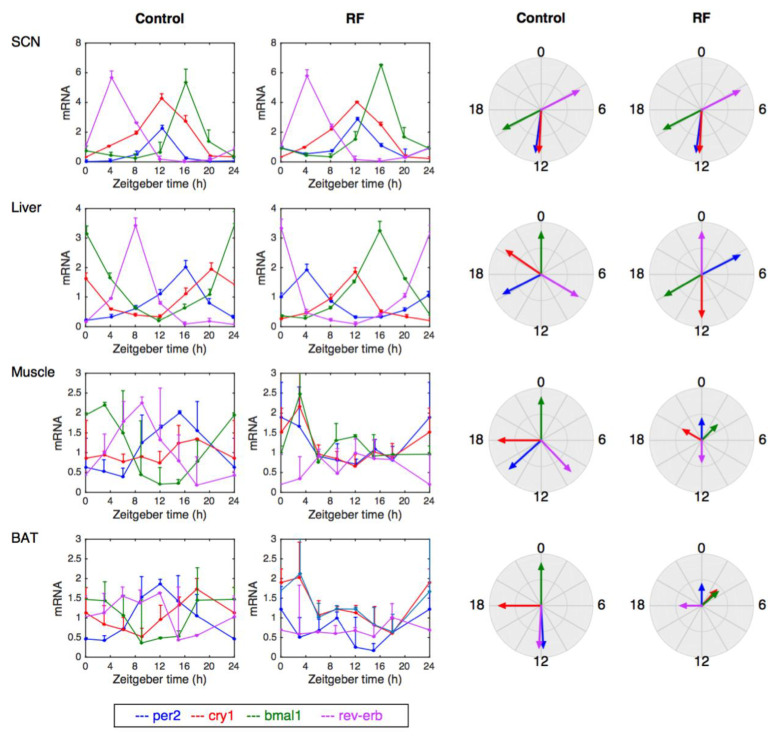
Experimentally measured clock gene expression in different organs in control (ad libitum) and daytime-restricted feeding (RT) conditions in rodents. Data for the suprachiasmatic nucleus (SCN) and liver are from [81]. Data for muscle and brown adipocytes (BATs) are from [84]. Note that for muscle and BATs, the data at t = 24 h are replicated from data at t = 0. The circle plots on the right show the phase of the peak of gene expression. The small size of the arrows for muscle and BATs under RT reflects the fact that the rhythmicity in these conditions is strongly altered. This figure highlights the organ-specific phase relationships between the expression profile of core clock genes and the gene-specific alteration of the expression profile under RT.

These observations are in line with the results of Opperhuizen et al. [83], who also showed that ill-timed feeding results in gene-specific phase shifts of clock and metabolic genes’ rhythms in the liver and in a loss of rhythmicity in the muscle in rats. Similarly, de Goede et al. [84] highlight differences in the gene expression in muscle and in brown adipose tissues upon time-restricted feeding in rats (Figure 2). These data support the hypothesis that time-restricted feeding leads to a desynchrony of peripheral organs from the SCN pacemaker and that the changes in clock gene expression are gene specific. Indeed, in a given organ, not all clock genes are affected in the same way, and the phase relationships between the genes are not conserved. In the following, we refer to these dysregulated clocks as “twisted clocks”.

In an earlier study, Bur et al. [85] compared the expression of clock genes in the liver and in the pituitary gland at different photoperiods and upon various feeding regimes in mice. The pituitary was chosen because it conveys the SCN signal to peripheral organs through periodic hormone release while being able to generate rhythmic expression of circadian genes independently of the SCN. The pituitary also controls the release of glucocorticoids (GCs) by the adrenal gland and, thereby, impacts peripheral clocks and metabolism. Bur and colleagues found that under the 12:12 LD cycle, the expression of clock genes was very similar in the liver and in the pituitary gland. Compared to the 12:12 LD cycle, oscillations under a long day (LD 16:8) appear delayed in a similar way in both organs. In contrast, under a short day (LD 8:16), the two organs exhibit different and gene-specific phase shifts (with respect to the 12:12 LD case). Bur et al. [85] also found that daytime-restricted feeding (compared to nighttime-restricted) reversed the rhythmicity of clock gene expression in the liver, whereas it strongly dampened the oscillations in the pituitary gland. These authors further noticed that, in the absence of GCs (adrenalectomized mice), the inversion of gene expression in the liver clock following the transition from day-restricted feeding to night-restricted feeding is faster (consistent with the hypothesis that the GCs acts as an inhibitor of phase shift), but that the daily expression of clock genes is not affected by the feeding timing. In contrast, GC removal does not alter the response of the pituitary clockwork to daytime-restricted feeding. The pituitary clock appears resilient to adrenalectomy. From the complex, intermediate response of the pituitary, the authors concluded that this gland integrates both light and food signals, contrarily to the SCN, which primarily adjusts to the light signal, and peripheral organs (such as the liver), which are mainly entrained by food.

The genetic and metabolic consequences of ill-timed feeding was further investigated by Mukherji et al. [81,82]. These authors found that transferring mice from nighttime to daytime feeding shifted the phase of metabolic markers (levels of blood glucose, insulin, free fatty acids, etc.) and that clock gene expression in peripheral organs was inverted, in contrast to the SCN pacemaker, which remains unaffected by the feeding schedule (Figure 2). Due to this desynchrony of the circadian system, animals developed metabolic syndrome such as hypoinsulinemia, hypertriglyceridemia, and hyperglycemia, which, in the long run, translate into pathologies including obesity and diabetes.

### 5.1. Internal Twist Explained by Mathematical Modeling 

**Figure 3:**
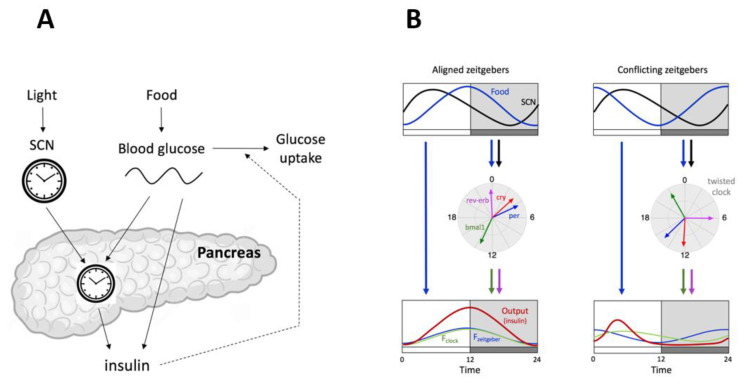
(**A**) Scheme of the model proposed by Woller and Gonze [86]. The release of insulin by the pancreas is controlled by the pancreatic circadian clock and by the level of circulating glucose, the latter being directly dependent on feeding. The pancreatic clock is controlled both by the light-dependent SCN clock and by the blood glucose. (**B**) Summary of the results obtained with the model [86]. In normal conditions, when light and food cues are aligned, the clock optimally mediates the response so that the output (insulin) reaches a high level when needed to store glucose. Under conflicting conditions, the expression of clock genes is altered (twisted clock), and this translates into an impaired output (phase shifted and low level of insulin rhythm and hyperglycemia).

A closer look at the time profiles of blood glucose, insulin, and the expression of clock genes reveals that the rhythms in the pancreas are not simply inverted upon inversion of the feeding time [81,82]. There is indeed a gene-specific phase shift in clock gene expression: whereas *Per2* is shifted by 12 h, *Bmal1* and *RevErbα* are shifted by 8 h. Such a twist would not be expected if the clock would be under the control of a single zeitgeber. In that case, the timing of the zeitgeber would simply dictate the phase of the clock, and the phase relationships between each gene would be set by the internal transcriptional–translational circuitry. Differential phase shifts can however be explained by the mismatch between two different signals entraining the peripheral clocks, one from the (unaffected) light-controlled SCN pacemaker and the other from the (inverted) nutrient cues [86]. More specifically, the conflicting inputs are likely to differently affect distinct core clock genes, and mathematical modeling has shown that this is sufficient to create an internal twist between these clock genes. These conflicting inputs could be, on the one hand, the nutrient sensors AMPK/SIRT1 and hormones (e.g., insulin), which mainly affect the negative limb of the clock [18,23,24,25,26] and on the other hand, cues from the autonomic nervous system, which seem to strongly affect the *Bmal1–RevErbα* loop [87]. In turn, this twist will affect downstream metabolic pathways. Indeed, since insulin secretion is under the control of both the pancreatic circadian clock and food-dependent blood glucose, its rhythm is not simply shifted but also reduced in amplitude, as supported by a mathematical model that incorporates both the nutrient periodic signal and the pancreatic circadian clock, which receives both the SCN and the nutrient cues (Figure 3) [86]. This altered rhythm in insulin impairs glucose regulation, such that the amplitude of glucose is increased (hyperglycemia), a model prediction consistent with the observations of Mukherji et al. [82].

Thus, once challenged by conflicting zeitgebers, the complex architecture of the circadian clock system is subject to twists in gene expression and, consequently, to metabolic disorders. We may wonder what the physiological advantage of such a complex and sensitive circadian architecture is. Some insights can be provided by mathematical modeling. The above model can indeed be modified to compare the original system with hypothetical alternative architectures, which either do not incorporate the influence of the SCN on the pancreatic clock or do not rely on any clock control [86]. Numerical analysis of the three systems predicts that in the absence of light information from the SCN, the pancreatic clock (and therefore glucose metabolism) would perfectly adapt to the new feeding schedule (no twist), but the adaptation of such a system is predicted to take some time because of the inertia of the local clock. In contrast, in the absence of any clock control, the metabolic response to large chronic changes in food availability is immediate, without any anticipation. This suggests that there is a tradeoff between the ability to sense light and nutrient cues (with anticipation) and the ability to fully adapt to chronic perturbations in these zeitgebers [86].

### 5.2. Another Conflicting Zeitgeber Paradigm 

In a recent work, Heyde and Oster [88] designed another conflicting zeitgeber paradigm. Metabolic activity was measured as well as clock gene expression in the SCN and in peripheral organs in mice subject to zeitgeber desynchrony. In one series of experiments, they apply a 28 h LD cycle (LD 14:14) and a 24 h feeding/fasting alternance (ZD condition). In a second series of experiments, these conditions are inversed: LD 12:12 and feeding/fasting 14:14 (inversed ZD or iZD condition). These protocols lead to an alternance of in-phase and out-of-phase regimes over time. Under ZD, most mice exhibit a period between 24 and 28 h in their locomotor activity, highlighting a combined effect of the two periodic cues on the overall circadian rhythmicity. Regarding clock gene expression, *Bmal1* and *Per2* profiles in the SCN are affected by the regime: both genes display a phase shift of about 5 h between the in-phase and out-of-phase regimes, but the amplitude of *Bmal1* expression appears weaker in antiphase days. The profiles of the clock gene expression in the liver, white adipocytes, and adrenal gland are locked to the feeding regime and less affected by the in-phase vs. out-of-phase condition. This is consistent with the fact the SCN clock genes are directly targeted by light input, whereas in these peripheral organs the expression of clock genes is mostly influenced by the feeding pattern. The release of corticosterone—the main GC in rats and mice—by the adrenal gland is slightly shifted and damped on antiphase days, possibly explained, at least partly, by the reduced expression of *Per2* in this condition. This impaired rhythmicity is expected to affect entrainment of the peripheral clock (which is known to be mediated by corticosterone). Leptin release, normally regulated by feeding, appears also to be influenced by the SCN under conflicting cues. Finally, mice under ZD or iZD undergo cycles of weight gain and loss without overall weight increase. This supports the hypothesis of a stabilization of peripheral clocks by the entrained SCN, allowing energy homeostasis, in healthy (in phase) conditions and a desynchrony in perturbed (out-of-phase) conditions. Mathematical modeling of such nontrivial dynamics would certainly be helpful to better grasp the internal logic of the circadian network.

## 6. Jet Lag and Gene-Specific Resynchronization Time

After a jet lag, the circadian system does not instantaneously synchronize to the new time frame. It takes some time for the molecular clock, and hence for the physiological processes, to adapt to the new schedule. This resynchronization time was shown to be tissue-specific and to be responsible for the temporary disruption of the overall physiological coordination. Yamazaki et al. [89] have found that during jet lag, *Per1* expression rhythm re-entrains faster in the SCN than in peripheral organs (skeletal muscle, liver, and lung), consistent with the fact that the LD cycle is the most important zeitgeber for the SCN. This difference in the resynchronization time also indicates that, while the SCN circadian pacemaker entrains circadian oscillators in the periphery, this inter-organ coupling is impaired after a jet lag. Similar conclusions were reported for *Per2* [90]. 

By measuring the expression of individual clock genes upon jet lag, Reddy et al. [91] and Kiessling et al. [92] found that the resynchronization time is gene specific. In the mouse SCN, in response to a large advance of the light–dark cycle, *Per* genes were found to resynchronize much faster than *Cry* genes, which readapt more gradually, similar to the activity–rest cycle [91]. Still in the SCN, Kiessling et al. [92] observed that the resynchronization time of the *Per* genes is much shorter than that for *Bmal1* or *RevErb*. Other transient dynamics are, however, observed in other organs. For example, in the pancreas, all genes take more time to re-entrain, and *RevErb*α appears to be slightly faster. 

This prompted the authors to have a closer look at the link between the pacemaker and the peripheral clocks. Adrenal glucocorticoids (GCs) are able to reset peripheral clocks [18], and the adrenal circadian clock regulates the rhythmic release of GCs into the blood [93]. Kiessling et al. [92] discovered that genetic ablation of the adrenal clock accelerated the rate of re-entrainment and that the application of an inhibitor of GC synthesis resulted in either acceleration or deceleration of behavioral adaptation to the new time zone, depending on the time at which the GC inhibitor is administered. They concluded that the adrenal circadian clock, through control of GC rhythms, is a major regulator of re-entrainment to jet lag.

**Figure 4:**
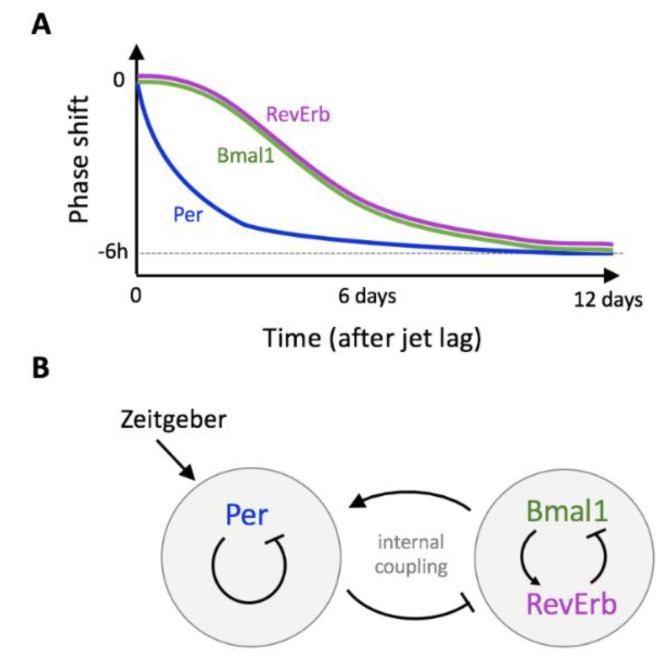
(**A**) Upon jet lag, not all clock genes resynchronize at the same speed [92,94]. (**B**) This transient twist can be explained by the internal desynchrony of the clock genes network [95].

In the same line, Ono et al. [94] reported that *Per* and *Bmal1* re-entrained at the new phase at different speeds after the application of a 9 h light pulse given just before the subjective night (Figure 4A). Such a dissociation between the *Per1* and *Bmal1* oscillations was observed in the SCN ex vivo and in living mice.

A strong heterogeneity in the kinetics of re-entrainment is thus found both at the tissue and at individual gene levels. These results suggest that the coordination of clock gene expression is globally disrupted during jet lag, creating a temporary twist of the local clocks. This likely explains why metabolic and activity rhythms are temporarily altered after a jet lag.

### Temporary Twist and Internal Decoupling Explained by Mathematical Modeling

Two hypotheses have been put forward to explain the observations: desynchronization at the single-cell level or emergence of two groups of cells in which either *Per* or *Bmal1* is predominant [94]. A surrogate data analysis of the experimental time series published by Kiessling et al. [92] supports the first hypothesis [95]. Schmal et al. [95] designed a conceptual mathematical model to show how internal dissociation can result from the decoupling between two autonomous oscillatory circuits (Figure 4B). This model is based on the hypothesis that *Per* and *Bmal1*/*Rev* intracellular regulatory feedback loops can independently generate oscillations. After calibration of the model to reproduce experimentally measured resynchronization times after a jet lag, they estimated the intrinsic period of each oscillator as well as its coupling strength. They also successfully reproduced these results with a minimal model and with a more detailed molecular model. The desynchronization of the *Per* and *Bmal1*/*RevErb* dynamics is obtained provided that *Per* and *Bmal1*/*RevErb* submodels can be dissociated. The latter model allows to link the coupling strength to an actual regulatory process, namely the strength of inhibition of *RevErb* transcription by PER protein. Modeling thus constitutes a valuable tool to interpret experimental data and to reveal design principles of the molecular architecture. The next step will be to understand how such an internal twist affects circadian outputs, such as the release of hormones and the metabolic activity.

## 7. Concluding Remarks

The observations reviewed in this paper, together with many others, highlight the importance of considering the circadian regulatory system not as a pure hierarchical system but rather as an intricate network of interconnected organs, each of them having its own clock, its own zeitgebers, and its own clock-controlled outputs. There is thus a need to examine gene expression at the level of each organ and to carefully characterize the profile of expression of each individual clock gene. Knockout studies are crucial to determine the role of each gene in the gene regulatory network. It is also important to complement these findings with studies that mimic natural—but possibly perturbed—conditions such as ill-time feeding or jet lags. Those conditions can induce gene-specific phase shift (“twist”) with potential negative consequences on energy homeostasis and physiology.

Since the discovery of the first clock genes, mathematical models for the core regulatory circuit have been developed. In parallel with the experimental discovery of additional genes and regulations, these models were updated and gained in complexity [63,64,65]. Once they account for the main features of the circadian oscillations and for the behavior of knockout mutants, these models can be used as a tool to interpret the experimental observations, which may defy intuition [96]. As exemplified in the present review, the gene-specific phase shift induced by time-restricted feeding and its consequence for the profile of glucose and insulin was successfully reproduced by a model that incorporates multiple inputs on the genetic clock [86]. The gene-specific resynchronization time after a jet lag was convincingly explained by an internal desynchronization of the gene clockwork [95]. Another recent theoretical study focused on the durations of the different circadian phases (defined by the elevated expression of given clock genes) and their alteration upon mutations [97]. Interestingly, this model highlights another possible type of twist. Indeed, the increase of the duration of the different phases upon mutation was found to be gene dependent: while the profile of some genes appears relatively insensitive to mutations, other genes appear highly expressed during a shorter time.

These theoretical works thus allow the exploration of the fine-tuning of the genetic circadian clocks, which in turn may be responsible for subtle changes in clock-controlled processes. Models focusing on the circadian control of metabolism already exist [98,99,100]. The next step will be to incorporate both genetic details and clock-controlled processes into an integrated model. We expect that in the future, with the accumulation of organ-specific measurements and high-resolution gene-specific expression recordings, quantitative computational models will play an important role in understanding how the complex circadian clock system integrates multiple environmental cues to guarantee a proper adaptation of physiology and metabolism and how misalignment causes diseases. This will open further perspectives to find new drugs and to establish new chronopharmacological protocols to treat these diseases.
